# Accreditation and professional integration experiences of internationally qualified dentists working in the United Kingdom

**DOI:** 10.1186/s12960-021-00703-y

**Published:** 2022-01-10

**Authors:** Latha S. Davda, David R. Radford, Sasha Scambler, Jennifer E. Gallagher

**Affiliations:** 1 University of Portsmouth Dental Academy, Faculty of Science and Health, University of Portsmouth, PO1 2QG Portsmouth, United Kingdom; 2 Faculty of Dentistry, Oral & Craniofacial Sciences, Centre for Host Microbiome Interactions, King’s College London, Denmark Hill Campus, SE5 9RS London, United Kingdom

**Keywords:** Professional integration, Overseas registration examination, Internationally qualified dentists, Oral healthcare professionals, Dental care professionals, United Kingdom, National Health Service/NHS

## Abstract

**Introduction:**

Regulatory processes for Oral health care professionals are considered essential for patient safety and to ensure health workforce quality. The global variation in their registration and regulation is under-reported in the literature. Regulatory systems could become a barrier to their national and international movement, leading to loss of skilled human resources. The General Dental Council is the regulatory authority in the UK, one of the nine regulators of health care overseen by the Professional Standards Authority.

**Aim:**

The aim of this paper is to present the professional integration experiences of internationally qualified dentists (IQDs) working in the UK, against the background of regulation and accreditation nationally.

**Methods:**

Registration data were obtained from the General Dental Council to inform the sampling and recruitment of research participants. Semi-structured interviews of 38 internationally qualified dentists working in the United Kingdom were conducted between August 2014 and October 2017. The topic guide which explored professional integration experiences of the dentists was informed by the literature, with new themes added inductively. A phenomenological approach involving an epistemological stance of interpretivism, was used with framework analysis to detect themes.

**Results:**

Internationally qualified dentist’s professional integration was influenced by factors that could be broadly classified as structural (source country training; registration and employment; variation in practising dentistry) and relational (experiences of discrimination; value of networks and support; and personal attributes). The routes to register for work as a dentist were perceived to favour UK dental graduates and those qualifying from the European Economic Area. Dentists from the rest of the world reported experiencing major hurdles including succeeding in the licensing examinations, English tests, proving immigration status and succeeding in obtaining a National Health Service performer number, all prior to being able to practice within state funded dental care.

**Conclusion:**

The pathways for dentists to register and work in state funded dental care in UK differ by geographic type of registrant, creating significant inconsistencies in their professional integration. Professional integration is perceived by an individual IQD as a continuum dictated by host countries health care systems, workforce recruitment policies, access to training, together with their professional and personal skills. The reliance of the UK on internationally qualified dentists has increased in the past two decades, however, it is not known how these trends will be affected by UK’s exit from the European Union and the COVID-19 pandemic.

**Supplementary Information:**

The online version contains supplementary material available at 10.1186/s12960-021-00703-y.

## Introduction

Most health professionals across the globe have to register with their respective national regulatory bodies and be licensed to work as a nurse, doctor, dentist or pharmacist [1]. These processes are considered essential for patient safety and maintaining standards of care. The wide geographical and intra-professional variation globally on the accreditation and regulation of oral health care professionals (OHCPs) including dentists is under-reported in literature [2]. Moreover, regulatory systems, particularly if protracted, could act as a barrier to the international movement of dentists, leading to loss of skilled human resources from the global workforce [3].

In dentistry globally, primary dental education is provided by multiple public and private sector universities and colleges and the regulating bodies may or may not be involved in accreditation of these health programmes [4]. In the UK, the General Dental Council (GDC), regulated by the Professional Standards Authority, is involved in accreditation of the primary dental education programmes across the four nations; and, as such, all dental professionals who graduate from UK schools may apply to register with the GDC to enable them to practise in their discipline of qualification. In the UK dentists with appropriate additional education, training, skills, and experience can additionally register as specialists in thirteen dental specialties [5].

Internationally qualified dentists (IQDs), defined as those dentists who have a primary qualification from outside the UK, intending to work in the UK can apply for registration in the UK, through one of the following four routes/pathways:

- Obtain a UK dental qualification
- Have a European Economic Area (EEA) countries’ qualification
- Qualify through the International qualifying examination (IQE) or Overseas registration examination (ORE) conducted by the GDC
- Others qualifying from countries with whom UK has bilateral agreements, where applications are judged on individual criteria

Based on the four routes described above, in 2019, there were 30,463 (72%) UK qualified dentists, and those qualifying outside the UK were 12,007 (28%). Among those qualifying outside UK, the figures were made up of 6,881 (16%) EEA qualified, 3,591 (8%) ORE qualified dentists and 1,535 (4%) other overseas qualified dentists [6]. Although, over the past 20 year period (2000–2019), there has been a net increase of 11,144 dentists registered with the GDC, over half of this number (*n* = 6416) were internationally qualified. This increase has led to changes in the composition of the dentist workforce providing dental care in UK (Additional file 1).

**Fig. 1:**
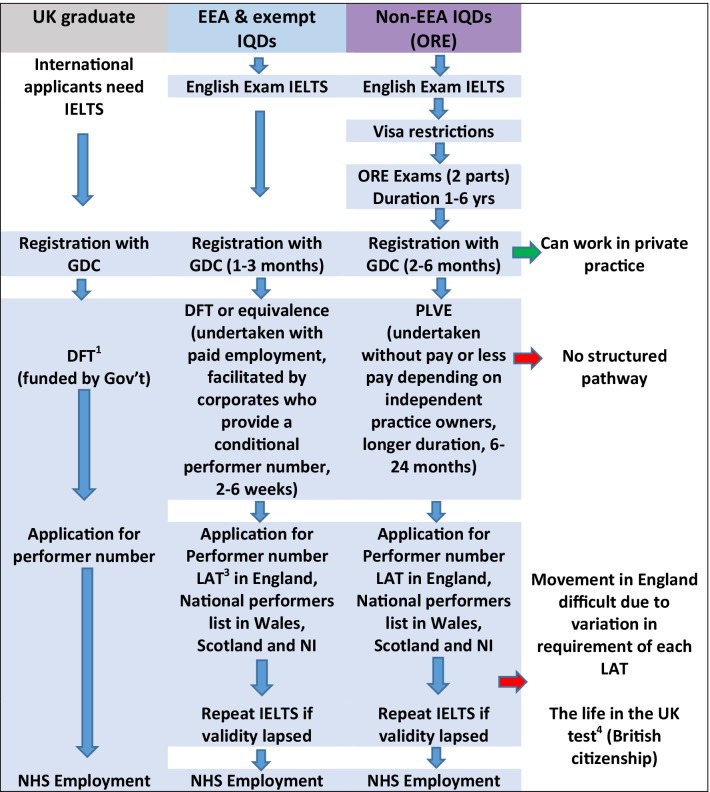
Routes to registration and employment for dentists in UK. (^1^DFT is dental foundation training which is compulsory for UK graduates to obtain a performer number to work in the NHS, EEA IQDs can compete for these posts, but cannot obtain employment without it in the NHS. ^2^PLVE—‘Performer List by Validation of Experience’ is a route for non-EEA dentists to apply for a performer number. ^3^LAT are the Local Area Teams. ^4^The life in the UK test [18] is a necessary test before application is made for British citizenship and examines both the English language and knowledge of British culture)

Oral disorders are amongst the leading burdens of untreated disease globally leading to poor quality of life [7]. This was attributed to various causes including a lack of dentists in some countries, migration of dentists to high income countries, inequitable distribution of dentists, their urban concentration resulting in a lack of access amongst rural population, and reluctance to work in the state commissioned dental services along with a mismatch of dental education to the disease profiles in the countries [8–10]. UK is a destination country for migrant dentists from Europe and outside Europe, potentially draining these countries of their highly skilled oral health workforce [6]. Similar to global trends, in the UK, there has been an increase in the migration of dentists from the NHS to private dental care [11], from rural to urban areas making access to NHS dentistry a concern with the public [4, 12, 13]. Recruitment of IQDs to work in the areas where there was lack of dentists or in secondary care especially in maxillofacial surgery departments in remote areas is not new, however, the increase in the number of IQDs since 2000 has raised concerns on ethical recruitment, competency of these migrant dentists and hence patient safety and the human rights of the IQDs [14–17]. The process for registration and employment in the National Health Service (NHS) suggests a variation for each registrant group and this may impact their professional integration experiences working in the UK (Fig. 1).

It is not known if the migration motivation and/or IQDs lack of access to the traditional training pathways and employment, influenced their professional integration in the destination country. Whilst, deskilling, career stagnation, lack of equal opportunities have been reported amongst/by international doctors and nurses migrating to the UK [14], there are no studies reporting the professional integration experiences of IQDs working in the UK. The aim of this paper is thus to explore the accreditation and professional integration experiences of internationally qualified dentists working in the UK. This will allow international comparisons to be made in future.

## Methods

Registrant profiles from the GDC [6], informed study sampling. The purposive sample included IQDs migrating from 25 countries (9 EEA, 16 non-EEA) spanning the six World Health Organization (WHO) regions. IQDs were registered with the GDC through one of the above four listed route. They were working part-time/full time across a range of settings (NHS/private general practice, NHS hospitals, dental schools and in community dental services) across the four nations of the UK. Participants were recruited through key gatekeepers including, educational bodies involved in training international dentists, Local Dental Committees, Clinical Directors of Corporate dental providers and through snowball sampling. Non-registered IQDs living in UK, in full time education and/or waiting to sit the licensing examination were excluded. Written consent for the interview followed the provision of information on how the data would be anonymised, its storage and dissemination through publications and the opportunity to ask questions about the study. A total of 38 semi-structured interviews were conducted between August 2014 and October 2017, of IQDs working in the UK. A phenomenological approach involving an epistemological stance of interpretivism, was used with framework analysis [19]. This approach was used as little was known about IQDs’ professional integration experiences in the UK. Ethical approval was provided by King’s College London Ethics Committee (Reference BDM/12/13-122). Participation in the research was voluntary and the primary researcher (LD) conducted pilot interviews to refine the interview technique. Names of people, organisations and other confidential information which may have been divulged during interviews was redacted from the transcripts to anonymise the data, prior to coding and analysis. Participants were identified by a number, gender, and the WHO region they qualified from, in order to protect their identity whilst providing insight to their background.

The interviews were conducted via telephone or face to face. Interviews lasted between 32 and 87 min (average 48 min); all interviews were recorded, transcribed verbatim and anonymised for analysis. Participants were asked about their dental education and work experience in the country they qualified, why, when and how they decide to migrate to the UK, what their experience of the registration process and obtaining employment was and their perceptions of working in the UK. Throughout the interview, the thoughts and opinions expressed by the participants were summarised and re-iterated to test and confirm the data. Thematic content analysis was carried out using the framework approach [19]. This included familiarisation by immersion in the raw data (listening to the recorded interviews repeatedly), constructing a thematic framework by coding the transcripts, mind mapping and sorting the data by themes, indexing or sorting to finalise coding framework (inductive coding initially conducted by LSD using Nvivo10^©^ and was discussed with other authors). Analysis was completed with the finalised list of codes applied to all interviews; mapping and interpretation helped to create descriptive accounts and typologies, while ensuring that the transparency of original data was maintained [19].

### General characteristics of the participants

In total 38 participants were interviewed, using a sampling matrix informed by the GDC registrant data. The matrix guided the selection of the IQDs to include participants based on gender, WHO regions where they migrated from, routes to registration, age at migration, and the region and type of current work in the UK (Table 1).

**Table 1:** Summary of demographics of the participants

Variable	Categories	No. of participants		
		Total	EEA*	Non-EEA
Gender	Male Female	18 20	7 7	11 13
Age at the time of interview	20–30 years 31–40 years 41–50 years Above 51 years	4 15 17 2	4 6 3 1	0 9 14 1
Age at the time of migration	18 years and below 19–30 years 31–40 years 41–50 years Above 51 years British born	1 23 9 2 1 2	1 8 2 2 0 1	0 15 7 0 1 1
Source country based on WHO regions	European Region^1^ South–East Asian Region Eastern Mediterranean Region African Region Western Pacific Region Region of the Americas	17 10 5 3 2 1	14 0 0 0 0 0	3 10 5 3 2 1
Route to registration with the General Dental Council (UK’s registering body)	IQE/ORE Exempt persons [EEA/Bilateral agreement^1^ None^2^	19 15 4	0 14 0	19 1 4
Current Status with the General Dental Council	GDC registered as dentists GDC registered as DCP GDC registered + SC (Dual registration) Not registered	32 1 2 3	14 0 0 0	18 1 2 3
Destination country in UK	England Wales Northern Ireland Scotland	33 2 2 1	12 0 1 1	21 2 1 0

*EEA-European Economic area

^1^The WHO Europe includes 53 countries in the continent of Europe, whilst the European Union (EU) was an economic and political union of 28 countries and European Economic Area (includes all EU countries and Norway, Iceland, Lichtenstein who have similar rights of access but are not EU members). At the time of research, the UK was still part of the EU, but had voted to leave in 2016

^2^Four IQDs were not registered with GDC; three were working in education, research and trade related to dentistry and one IQD was registered as dental therapist

**Fig. 2:**
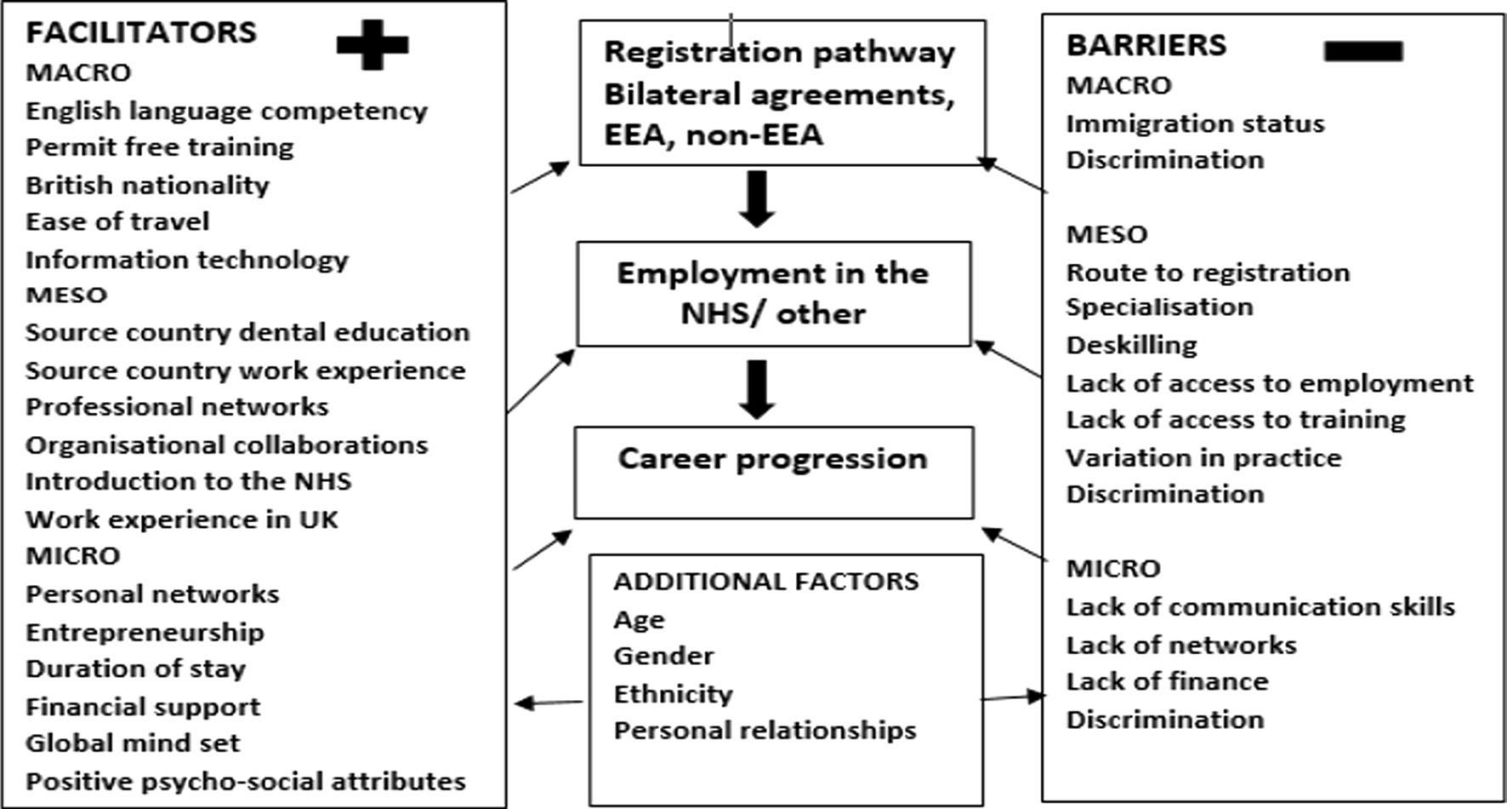
Facilitators of and barriers to professional integration among IQDs working in the UK

The facilitators of, and barriers to, professional integration against which the IQDs reported their experiences could be grouped as macro factors (experiences linked to national and regional policies), meso factors (experiences directly linked to the practice of dentistry) and micro factors (experiences linked to the personal status, knowledge, skills and ability of individual) (Fig. 2). Their professional integration experiences were further compounded by age, gender, ethnicity and personal and professional networks.

## Results

### Professional integration experiences of IQDs

Professional integration appeared to be a dynamic process, which was linked with the respondent’s personal and professional identity and changed during their stay in the UK. The way they interpreted and responded to the structural challenges they faced, using their network support mechanisms and their personal attributes, ultimately affected career aspirations and progression. IQD’s in this study reported that their professional integration was influenced by factors that could be broadly classified into *six themes*; the first three relate to the structural context and the other three are relational (Table 2).

**Table 2:** Themes derived from the qualitative data

Structural themes The expectations of the IQDs before migration, the time lapse from migration to becoming employed in the UK and the work experiences thereafter were directly influenced by their country of primary qualification (EEA, non-EEA), immigration status and routes to registration	Relational themes These themes spanned across the narratives and all stages of their integration journeys, irrespective of their migration motivations, facilitators of and barriers to professional integration
Source country training Registration and employment Variation in practising dentistry	Experiences of discrimination Value of networks and support Personal attributes

### Source country training

The length of undergraduate dental education in source countries varied (4–6 years), followed by a year of work experience (internship). There was variation in IQDs’ reported confidence in their ability to undertake various dental procedures when they qualified. Differences in the curriculum, mismatch of skills acquired during training and their application in the UK, and a lack of knowledge on how dentistry operates in the NHS were common to both EEA and non-EEA IQDs.

“We did get a lot of experience in extractions and fillings, but we didn’t have any experience on full root canal treatment... we just did one or two bridges, very limited.”(45 yrs, Female, Eastern Mediterranean region)

“so one thing is the NHS that I had to get used to, I found it hard to learn the system.”(36 yrs, Male, European region)

While ‘brain drain’ is considered an important issue in health professionals’ migration [20–23], on an individual level, IQDs did not think of their migration as ‘brain drain’.

“I don’t because … I should be contributing to that society (where I live) rather than boundaries (imposed by country where you were born).”(45 yrs, Female, Eastern Mediterranean region)

In the above example, the dentists’ acquired skills were simply being utilised in a different geographical context.

### Registration and employment experiences

The barriers faced by the IQDs included stringent registration examinations, English language competency and access to training pathways that led to employment in the NHS. Non-EEA IQDs had to overcome more barriers than EEA IQDs and those coming from countries with bilateral agreements (Republic of Ireland, South Africa before 2001). The experiences of the IQDs spanned several years, during which there were a number of changes to the registration, NHS employment and immigration policies. The process was quicker for EEA dentists.

“When I decided that it was time to move for me, ... I was working in EEA home country and then I sent all the documents to the GDC to look for my diploma recognition and everything. Therefore, they assessed everything after a few months, ...I started to apply for jobs as a dentist” (27 yrs, Female, European region)

For non-EEA dentists the process was reported as lengthy, primarily due to the challenges posed by the registration examination, such as the high cost, limited access to examination places, lack of clarity and perceived fairness of the process and the length of time it takes. The structure of the registration examination for IQDs has changed over the past 15 years and currently, the ORE has two parts: Part I consisting of a computer-based assessment on the knowledge in all areas of dentistry and Part II consisting of 3 sections; OSCEs, diagnosis and treatment planning and simulated dental exercises. Candidates have to pass each section and no compensation is awarded between sections. If they fail a section, they have to repeat the entire examination. These high stakes examinations were not held very frequently and the waiting times and booking system added to their anxieties.

“I’ll tell you when I think about this exam, I have nightmares… it was awful yes, … the prices of exams … and I had to do it three times.”(44 yrs, Female, American region)

Equally some IQDs found these examinations useful.

“…It had prepared me really well to work in UK, really practical oriented I felt.” (38 yrs, Female, South-East Asian region)

English language which acted as a macro migration factor attracting health professionals from English speaking countries, also influenced their professional integration. The regulations around the English language testing applies to all dental health professionals who wish to work in the UK with set threshold requirements by the GDC.

Once registered, in order to work in the NHS, dentists are required to have a performer number which was issued previously by the Primary Care Trusts (until April 2013) and later on by the Local Area Teams (LATs) (Fig. 1). It is compulsory for the UK dentists to do Dental Foundation Training (DFT previously called as Vocational training). On the other hand EEA IQDs could directly work in the NHS without undergoing DFT. However, Non-EEA IQDs had to go through a process called the Performer List Validation by Equivalence (PLVE). For non-EEA IQDs this prevented them from working in the NHS, even after being successful in obtaining registration. NHS practices naturally were reluctant to support IQDs as there were no financial incentives from the government or NHS to support them.

“Vocation training was the hardest one, so again. There was not many places, you’re competing against the local graduates, rich ones (as some IQDs paid the practice to be supported to obtain a performer number through PLVE) …but generally advertising and most times the place were kind of already … it is a fixed, you just go for interview, but they already appointed someone.”(38 yrs, Female, European region)

“Then after registration again finding the VT Equivalence was difficult. I went for many interviews but didn’t get it… (Finally) I got a job in XXX after 3 months... I got the performer number in 6 months.”(42 yrs, Female, South East Asia)

This resulted in exploitation of IQDs by some practices who extracted unpaid or low paid labour from the IQDs and charged the IQDs for supporting them through the PLVE application process. This further increased the IQDs financial hardships and alienated them further.

### Variation in practice in the UK

Once the IQDs were working some felt valued by their patients, colleagues, staff and organizations. Others reported variation in practice with regards to team dynamics, NHS regulations and the consent process. They reported fear to raise concerns and fear of litigation. Many reported satisfaction with the structured way NHS dentistry is delivered. Some IQDs liked the ethos of the NHS and the fact that they did not have to sell treatment to the patients.

“I hated the fact that I had to sell a treatment to somebody …it is not something I’m very good at. Whereas on the NHS… you don’t have to sell.”(35 yrs, Female, European region)

Many IQDs, who had worked less than five years in the UK, did not feel empowered to raise concerns with their employers. Those who worked in the UK for longer were more aware of their rights and felt confident to raise concerns and believed that any grievance will be investigated appropriately. IQDs were acutely aware of the possibility of litigation and complaints to the GDC.

“…the level of litigation and the amount of lectures we had that scared the life out of us about dentistry (laughs)…; basically, it is made out, it seem like everyone was out to sue you… don’t get me wrong, it is important to be defensive...but… Because of that I was just terrified of doing anything”. (26 yrs, Male, European region)

### Experiences of discrimination

Whilst discrimination in the country of qualification forced some IQDs to migrate, others reported experiencing discrimination in the UK mainly due to the variation in the registration process, lack of access to NHS employment and lack of access to training. This was perceived more strongly by the non-EEA IQDs which was not surprising considering the number of barriers they have had to overcome before they could practice.

“I think the worst thing, I feel unfair when I see Europeans dentists coming and they don’t do any exams and just easily get everything.”(38 yrs, Female, European region)

Discrimination in Academia was reported, where research done outside EU or UK was considered less robust and less valued.

“... when you’re reviewing a research paper and if you see it is from University that’s not got a good reputation for instance, one outside of EU … you might, … be a bit more critical of the paper.”(26 yrs, Male, European region)

British citizens who had travelled abroad for their primary dental qualifications were very vocal about the discrimination they faced, when they returned to practice in the UK. The registration process with the GDC was perceived as being discriminatory.

“Registering with the GDC was a painstakingly long difficult process. (IQD was a British Citizen) … I was made to do the IELTS exam, to show my level of English (in spite of completing secondary education in the UK).” (35 yrs, Female, European region)

Most of the IQDs, when first questioned, reported that they did not feel any discrimination from patients but on probing, few instances of discrimination based on the country of origin, gender, accents and ethnicity were reported. Many participants chose to ignore these and did not report these to management.

“So firstly patients, I have been told to go back to my own country. [Laughs].”(64 yrs, Male, African region)

“Some people are looking at you and are thinking, well you are a woman, I want to see a man... they tend to moan at reception, oh she's a foreigner, where is she from.”(35 yrs, Female, European region)

### Value of networks and support

Many IQDs reported that their families supported them financially, emotionally and socially before, during and after migration. Professionally, IQDs established informal social networks in real and virtual worlds, based on their country of origin and ethnicity. They used social media to help with their professional needs e.g., the ORE group on Facebook. These informal networks helped new migrants with information and guidance.

“If I can be truthful, there were quite a few support groups run by international dentists themselves, … those were really amazing, I don’t think I would have been able to do it without them …. But definitely none [sic support groups] of the formal bodies, no.” (31 yrs, Female, Eastern Mediterranean region)

After migration, the IQDs sought colleagues from their own country to navigate the processes and systems in UK. These networks were used mainly for social support, but some also used them for professional support. Some of them especially in major cities for example London, could work in a practice that was entirely staffed by IQDs.

“It is difficult because I didn’t have my performance number so I could only apply to private practices. I think I was lucky I met a few people that had practises and they gave me a hand. They were people from my country, they knew my background so they offered me the job.”(44 yrs, Female, American region).

“The good thing is that you have a lot of … people from your own school and your own college (from country they qualified from) here.”(44 yrs, Male, South East Asian region)

Younger IQDs, who had grown up in the digital world, however, did not report feeling isolated as they used social media to stay in touch with their families and believed that the distances were not great should they wish to travel due to improved connectivity (pre-COVID-19 pandemic).

“Yeah, it is really good... they call me or them text me, we sometimes talk through Skype or WhatsApp. So I feel when I was working in home country, I used to see them more than I see them (now)... but I mean, it is all the same.”(27 yrs, Female, European region)

Most IQDs reported not feeling supported by official organisations in the UK.

“…official bodies which are the first points of contact for people who come from abroad are very unaware on how to handle international dentists, where to guide them, where to direct them, what to do for them, so you end up wasting so much time trying to figure out who to speak to in the first place.”(31 yrs, Female, Eastern Mediterranean region)

### Personal attributes

Personal attributes of resilience, hardworking and perseverance were reported by IQDs in this study. Among the demographic factors identified, professional integration was mostly influenced by their source country of qualification, duration of work experience whether in the source country or in the UK and their gender. The source country was important as it influenced their knowledge and communication skills and hence the ease with which they negotiated the registration and employment process. Gender influenced professional integration with some female IQDs holding their careers back to enable childcare, support their spouse’s careers and children’s education. At the same time, women from the EMR region felt liberated and felt that they had achieved more professionally than they would have achieved in their source country. Information technology helped migration of EEA dentists as they could apply for registration and jobs online, thus saving resources and ensured that the transition was smoother before they migrated to the UK. A combination of knowledge of English and information available in English on the internet helped some IQDs to be well informed before migrating and this helped them integrate better.

“I did everything myself ...I was getting the information on the internet, was in English, ... Basically curiosity and wanting something new, to get the best for my life (resulted in me migrating).”(27 yrs, Female, European region)

## Discussion

Professional integration of participating IQDs who had successfully registered in the UK was affected by the structural processes involved in accreditation and regulation of dental professionals, recruitment in the NHS and immigration. The source country of qualification of the IQD appeared to determine the time taken to gain registration and hence professional integration. Whilst the process of securing employment was short (3–6 months) for dentists from EEA, and for those migrating from countries with bilateral agreements with UK, it was protracted (2–6 years) for non-EEA IDGs. These therefore emerged as structural themes. The relational themes of discrimination, value of networks and role of personal attributes spanned across their narrative of their professional integration journeys.

The different pathways for EEA and non-EEA qualified dental graduates created variation in the professional integration experiences similar to those reported among migrant doctors in UK [24]. The ‘Modernising NHS Dentistry Plan’ [25, 26], led to dental workforce reviews in the UK, during 2000 and 2004. Recruitment of IQDs was one of the policy measures implemented to increase dentist supply, the others being increasing UK graduate numbers and use of skill-mix [27, 28]. Other important events that facilitated IQD migration to the UK were: the expansion of the EU in 2004, 2007, 2013; the EU directive for mutual recognition of professional qualifications in the EEA region and the start of the overseas registration examinations [6, 29, 30]. The first two led to the supply of the IQDs from EEA region and the third provided a route for non-EEA IQDs to enter the UK.

Internationalisation of dental education [31], with a neo-liberalistic approach, further increased British citizens travelling to countries outside the UK to gain under-graduate dental qualifications (Education-tourist migrants) and this study found that these individuals faced challenges similar to those of IQDs who were not UK-born. This trend is similar to that seen in medical and nursing education in the past decade [32, 33]. This creates complex routes of migration [34], and bottle necks in the training pathways competing for the same career development places that are usually reserved for the domestic graduates. Non availability of internships and speciality training opportunities when they return to their country of birth is associated with difficulty in professional integration and career stagnation for these graduates.

The requirement for a registration examination in destination countries is usually centred on patient safety. Often ‘Fitness to practice’ data are used by the regulating bodies to raise this as a concern and justify processes in place. It is essential that this is discussed in the context of discrimination as concerns against international health professionals may therefore be raised by fellow professionals due to unconscious bias, lack of diversity and cultural insights into people behaviour [35, 36]. In reality, analysis of ‘Fitness to Practice’ data (7526 cases in 2017), revealed that EEA IQDs were involved in 30.9%, UK graduates 18.8% and non-EEA IQDs 0.4% of the cases. Interestingly, other UK trained dental care professionals such as nurses and hygienists were involved in 29.1% of the cases [37]. When the three most common subgroups of the complaints were analysed, those relating the failure to ‘communicate effectively’ was the same for both EEA and UK graduates, while EEA dentists had more complaints on ‘failure to put the patient’s interest first’ and ‘maintain and protect patients’[37]. The final outcomes showed that there was a higher proportion of sanctions against EEA dentists (12.8%) compared with UK dentists (7.3%). There is no formal research to better understand these data; possible reasons as reported among migrant doctors, could be deficiencies in the overseas professional’s clinical performance, biased reporting from staff and patients against them or the process of migration impacting their performance [24].

Most non-EEA IQDs and UK born dentists who qualified abroad reported feeling discriminated as a result of the structure of accreditation and recruitment processes. Discrimination was also reported by a few IQDs relating to gender, ethnicity, accent and country of origin. It is worth noting that instances of discrimination are often under reported by minority groups [38], thus, this may represent an under-representation of the problem. Migrant nurses have reported direct and indirect discrimination by staff, patients and organisations, whilst doctors reported feeling discriminated due to the lack of access to training and career progression [39, 40].

Discrimination in employment led to IQDs in this study to seek informal support amongst dentists from their own ethnic groups, who acted as informal mentors and provided guidance and even employment. This appeared to support and benefit IQDs from larger diasporas. An interesting type of clustering was evident among IQDs in some parts of the UK, such as Wales and in inner city locations, where dental practices were mainly staffed by IQDs, potentially suggesting that these posts or practice locations were less attractive to UK graduates. This type of ethnic and geographical clustering has been reported among South-Asian doctors who ended up in large numbers as consultants in the speciality of Geriatrics due to discrimination, lack of entry into other specialities or in Teaching hospitals [41]. Among the cohort of dentists participating in this study, difficulty in securing NHS posts in primary care, lead them to working exclusively in the private sector staffed by the respective ethnic groups sitting outside the state funded dental care. This, in combination with the geographical clustering of the dental practices in urban areas, could lead to rural populations and some urban areas with poorer populations not having access to NHS dental care, as more and more UK graduates choose not to work in the NHS, further exacerbating inequalities in access to oral health care and in oral health [42].

The value of professional and personal networks was a strong theme in IQDs professional integration. Whilst migrants have social capital, prior to migration, after migration they may additionally, establish links with the existing non-migrant populations. This sets them at a crossroad of cultures and some well-established migrants could act as bridging capital, influencing further migration [43]. Migration itself can create a new group or a new network that feel connected to each other by their very experience of migration as seen among IQDs [44]. In addition, vertical bonding with those who migrated previously can further strengthen migrants’ social capital [45]. Transnational family connections and alumni connections were evident in providing informal support to IQDs similar to those reported by doctors in previous studies [46, 47], and worked in similar ways according to “who one knew” rather than on merit. These networks require scrutiny in a manner similar to non-migrant networks [43]. This is of particular importance to dentistry as several independent dental practices are run as small family businesses in most source and destination countries. These routes were also beneficial to the IQD migrants in helping to avoid discrimination which they may have faced if they were to join larger practices or institutions. This may be one of the reasons that the IQDs in this study reported less discrimination once employed, unlike doctors and nurses [39, 48].

It was evident in this cohort of IQDs that some were constantly comparing themselves, not only to IQDs in this country, but also, to those peers they had left behind. This highlighted that those networks, both real and/or virtual, whether they were situated in source country/UK, that were based on a common purpose (IQE/ORE), ethnicity or country of origin were important for the IQDs in their overall professional integration and may potentially contribute to global knowledge transfer.

Personal attributes of resilience, hardworking and perseverance were reported by IQDs in this study. ‘Willingness to learn’ was attributed to the success and the ability of migrants to thrive in a new environment and this was found to be higher in migrant population than that of the native population in the technical sector [49]. Although such comparative conclusions cannot be drawn with the present data, it is essential that the migrant IQDs have the ability to overcome the numerous barriers in order to be successful and that is evident from this study.

Internationalisation of dental education, advances in information technology, and transnational networks have further facilitated the migration of dentists to the UK [50]. A global mindset and a culture of emigration observed in this study, has been reported in post-graduate trainee doctors in Ireland [51], as a necessity for career progression. Similarly, a dual culture of migration described among skilled health workers migrating from the Pacific Islands [52] was reported with families investing in facilitating migration of one family member to provide financial support in return. The reported outward migration from Ireland however, resulted on more reliance on a workforce from poorer countries to address the workforce shortage created by migration. Similarly, UK graduates formed the largest group of migrant dentists in Australia, whilst UK relied mainly on dentists from EEA to fill workforce gaps [53]. Recruitment and retention of dentists in the NHS is becoming increasingly difficult, as IQDs reported disappointment with NHS dentistry, fear of litigation and dislike of the burden of paperwork involved in general dental practice, which is similar to that reported by UK graduates. Similar sentiments were expressed by the EEA IQDs in a survey conducted by the GDC [54]. 24% of EEA IQDs responding to the GDC survey reported that the state of NHS dentistry was their main reason they were thinking of returning to their source country.

The UK’s departure from the EU in 2020, and the COVID-19 pandemic, have been paralleled by a decrease in the volume of overall migration for work and study in the UK and this may have impacted dentistry [55]. Considering that in 2020, 16% (*n* = 6966) of the dentists working in the UK were from the EEA, the process of withdrawal of the UK from the EEA potentially could impact the workforce supply from that source. The transition arrangements allowing EEA dental professionals to register with the GDC has been extended to 1st January 2023 [56]. The COVID-19 pandemic, has also resulted in indefinite suspension of registration examinations (ORE) for non-EEA dentists who form 3% (*n* = 1354) of total registrants, but with a decrease in the UK dental student intake; this could further decrease the dentists workforce [57, 58]. The indefinite suspension of ORE examinations in the UK in March 2020, following the COVID-19 pandemic, has left several IQDs facing the prospect of career stagnation, deskilling and loss of earning [57]. It is not yet known what the impact of this will be in the longer term, on these highly skilled migrants. The restrictions on international travel imposed by COVID-19 pandemic may reduce the number of students travelling abroad for education and health care workers may choose to ‘return migrate’ to be close to their families in times of crisis [59].

### Strengths and limitations of this study

This qualitative study gives a ‘voice’ to the professional integration experiences of the IQDs demonstrating the impact of the health systems and other regulatory policies on this highly skilled workforce. The 38 interviews have collected the experiences of a wide range of IDGs entering the UK through various routes, working for various durations in a range of settings and locations across the four countries of the UK. The participants were purposively drawn from the main supply countries that contributed the maximum number of IQDs to the UK in the last two decades. It contributes to the important wider research on global migration of health professionals and how this impacts health systems, with a focus on dentistry. The limitations were, that the sample included only those who were successful in working in the dental profession and those willing to talk and this may have led to self-selection of IQDs who may have strong opinions or grievances. The findings cannot be extrapolated to all IQDs working in the UK or to another timeframe. The potential bias in collection and analysis of the data with the primary researcher (LD) being an IQD, was recognised at the outset and overcome by ensuring strict adherence to the research protocol, following the sample matrix, methodical data collection, open and transparent data analysis and reporting.

## Conclusion

The UK has been an attractive country for international dentist migration over the past two decades. Nonetheless, migrating dentists face a range of challenges related to registration, immigration and social re-location that impacts on their professional integration. A better understanding of the barriers to and facilitators of professional integration in the UK, may help IQDs in their decision making before they migrate. It may also help policy makers in managing dentist migration more effectively.

## Supplementary Information

**Additional file 1. **Subthemes and main themes from the qualitative data.

## Data Availability

The data sets generated during and/or analysed during the current study are not publicly available due to the risk of identification as they include transcripts of interviews, but redacted versions are available from the corresponding author on reasonable request.
