# Supplementary material for: Accreditation and professional integration experiences of internationally qualified dentists working in the United Kingdom
Source: Hum Resour Health. 2022 Jan 10;20:7. doi: 10.1186/s12960-021-00703-y (PMC8744036; doi:10.1186/s12960-021-00703-y)
Supplement: Supplementary file 1 — Additional file 1. Subthemes and main themes from the qualitative data. [file 12960_2021_703_MOESM1_ESM.docx]

| **Subthemes derived from 38 interviews** | **Main themes** |
| --- | --- |
| **Dental education in source country**  Variation in curriculum, knowledge and skills learnt  Variation in knowledge and skills applied  Variation in professional conduct, ethics and team work  Access to Post graduate training or specialisation  **Work experience in source country**  Lack of experience in primary care  Deskilling to career breaks – unemployment, maternity break  Deskilling in primary care dentistry as they were working as specialists or working in education or research | **Source country experiences** |
| **Immigration experiences**  Changes to immigration policies  European Union regulations  UK’s exit from EU - Brexit  **Registration experiences**  Routes to registration with GDC  IQE/ORE experiences  English examination  **Employment experiences**  Deskilling due to lengthy registration and employment process  Access to Dental Foundation Training (DFT)  Access to NHS employment | **Registration and employment in the UK** |
| **Being valued**  Being valued by patients, colleagues, staff, organizations  **Team dynamics**  Attitude to the DCPs, colleagues  **Variation in practice**  Fear to raise concerns  Fear of litigation  **Career progression**  Accepting lower grades at entry level  Ability to train or gain skills needed to match the market  Access to postgraduate training  Change of career pathways  Entrepreneurship  Contributions to dentistry in UK, transit countries and source country  Career aspirations  Broader social, cultural, economic integration | **Practising dentistry in the UK** |
| **Organisational level**  Discrimination from organizations involved in registration, employment and training  Exploitation by employers  Discrimination in pay  Discrimination in progression  Selective recognition of qualifications and skills  **Individual level**  Discrimination from patients  Discrimination from colleagues  Discrimination from managers/consultants  **Attitude to discrimination**  Focus on long term goals  Working harder to achieve goals  Moving away from ethnocentric views  Resilience  Acceptance | **Discrimination experiences and attitude** |
| Family networks and mentors  Friends networks and mentors  Access to formal professional networks  Access to informal professional networks  Real and virtual networks  Networks based on common purpose, ethnicity, source country | **Networks and support** |
| **Demographic factors**  Age, Gender, Ethnicity  Country of qualification  Duration of time in the UK  **Psycho-social attributes**  Hard working  Resilience  Adaptability – willingness to learn  **Communication skills**  Fluency of English language  Language barrier affecting confidence  Good interpersonal skills- Verbal and non-verbal skills  **Technical skills**  Knowledge of the health system (specially the NHS)  Dental procedural technical skills  Information technology related skills | **Personal attributes** |
